# Cytokinin production by *Pseudomonas fluorescens* G20-18 determines biocontrol activity against *Pseudomonas syringae* in *Arabidopsis*

**DOI:** 10.1038/srep23310

**Published:** 2016-03-17

**Authors:** Dominik K. Großkinsky, Richard Tafner, María V. Moreno, Sebastian A. Stenglein, Inés E. García de Salamone, Louise M. Nelson, Ondřej Novák, Miroslav Strnad, Eric van der Graaff, Thomas Roitsch

**Affiliations:** 1 Department of Plant and Environmental Sciences, Copenhagen Plant Science Centre, University of Copenhagen, Højbakkegård Allé 13, 2630 Taastrup, Denmark; 2 Department of Plant Physiology, Institute of Plant Sciences, University of Graz, Schubertstraße 51, 8010 Graz, Austria; 3 Laboratorio de Biología Funcional y Biotecnología (BIOLAB)-CICBA-INBIOTEC-CONICET, Facultad de Agronomía de Azul-UNCPBA, Av. República de Italia 780, 7300 Azul, Buenos Aires, Argentina; 4 Cátedra de Microbiología, Facultad de Agronomía de Azul-UNCPBA, Av. República de Italia 780, 7300 Azul, Buenos Aires, Argentina; 5 Cátedra de Microbiología Agrícola, Facultad de Agronomía, Universidad de Buenos Aires, Av. San Martín 4453, Buenos Aires 1417, Argentina; 6 Department of Biology, Irving K Barber School of Arts and Sciences, University of British Columbia Okanagan Campus, 3333 University Way, Kelowna, BC V1V 1V7, Canada; 7 Laboratory of Growth Regulators, Centre of the Region Haná for Biotechnological and Agricultural Research, Institute of Experimental Botany ASCR & Faculty of Science, Palacký University, Olomouc, Czech Republic; 8 Global Change Research Centre, Czech Globe AS CR, v.v.i., Drásov 470, Cz-664 24 Drásov, Czech Republic

## Abstract

Plant beneficial microbes mediate biocontrol of diseases by interfering with pathogens or via strengthening the host. Although phytohormones, including cytokinins, are known to regulate plant development and physiology as well as plant immunity, their production by microorganisms has not been considered as a biocontrol mechanism. Here we identify the ability of *Pseudomonas fluorescens* G20-18 to efficiently control *P. syringae* infection in *Arabidopsis*, allowing maintenance of tissue integrity and ultimately biomass yield. Microbial cytokinin production was identified as a key determinant for this biocontrol effect on the hemibiotrophic bacterial pathogen. While cytokinin-deficient loss-of-function mutants of G20-18 exhibit impaired biocontrol, functional complementation with cytokinin biosynthetic genes restores cytokinin-mediated biocontrol, which is correlated with differential cytokinin levels *in planta*. *Arabidopsis* mutant analyses revealed the necessity of functional plant cytokinin perception and salicylic acid-dependent defence signalling for this biocontrol mechanism. These results demonstrate microbial cytokinin production as a novel microbe-based, hormone-mediated concept of biocontrol. This mechanism provides a basis to potentially develop novel, integrated plant protection strategies combining promotion of growth, a favourable physiological status and activation of fine-tuned direct defence and abiotic stress resilience.

Throughout their life cycle, plants interact with a multitude of environmental factors, including unfavourable abiotic stress conditions and threats from a wide range of insects and pathogenic microbes. Phytohormone signalling plays a crucial role in accurately regulating plant responses. Ethylene (ET), jasmonic (JA) and salicylic acid (SA) are essential phytohormonal regulators of plant immunity that form a central signalling backbone which specifically coordinates defence responses against biotrophic and necrotrophic pathogens^1^. Detailed analyses of phytohormone function in plant immunity have extended this network to other classic growth-regulating phytohormones such as abscisic acid (ABA), auxins and gibberellins^2^,^3^,^4^. The classic growth-stimulating phytohormone family of cytokinins (CKs) comprises important regulators of many physiological and developmental plant processes such as cell division, leaf senescence, nutrient mobilization, apical dominance, and seed germination^5^,^6^. In the interaction of plants with insects and microbes, CK alterations have been identified to cause green island formation, galls, growth abnormalities^7^, and modulation of primary carbon metabolism^8^. As they induce sink metabolism^7^,^9^, CKs have been suggested to alter host physiology to facilitate maximum access of (hemi)biotrophic pathogens to nutrients during early interactions^7^. However, recently, significant direct functions for CKs in plant immunity have been identified in different plant species such as *Arabidopsis thaliana*^10^,^11^, tobacco^12^, and rice^13^ via induction of resistance against primarily (hemi)biotrophic pathogens such as *Pseudomonas syringae* and *Hyaloperonospora arabidopsidis* or by activation of defence responses (independent of pathogen infection). The underlying mechanisms mediating CK-dependent resistance against *P. syringae* include induction of SA in *Arabidopsis* and tobacco^10^,^12^, induction of phytoalexin accumulation^12^,^14^ and reduction of ABA levels in tobacco^15^. Furthermore, CKs were demonstrated to induce defence gene expression synergistically with SA^13^ and to enhance diterpenoid phytoalexin accumulation^16^ in rice.

In addition to pathogens, plants interact with a multitude of beneficial microbes, many of which belong to the genera *Azospirillum*, *Bacillus* or *Pseudomonas* and are characterized by their ability to promote plant growth, increase tolerance to environmental stress and/or enhance disease resistance. Agricultural food production faces many challenges due to increasing world population, climate change and restrictions on use of classic pesticides. Consequently, alternative plant protection strategies are urgently required. The biological control of plant diseases by beneficial microbes offers significant potential for integrated plant disease management^17^. To facilitate the development of microbe-based biocontrol strategies, their underlying mechanisms have to be fully elucidated. Known biocontrol mechanisms include (i) direct interference with the pathogen, such as competition for nutrients and space, secretion of antibiotics or degradation of virulence factors, and (ii) the induction of host plant resistance, which is often related to induced systemic resistance (ISR) involving the phytohormones ET and JA^18^,^19^,^20^. Interestingly, beneficial microbes are capable of producing different phytohormones, notably including CKs. Therefore, it is intriguing that CKs exhibit similar biological effects as described for beneficial microbes including the induction of plant growth promotion (PGP), environmental stress tolerance and disease resistance. Despite this correlative evidence, microbial phytohormones - and particularly CKs - have not been considered as a determinant for effective biocontrol of plant diseases. Microbial CK production has so far only been linked to PGP^21^,^22^ and suggested as a mechanism for increasing abiotic stress tolerance in plants^23^. Considering the widespread CK production by beneficial microbes and recent advances in understanding CK function in plant resistance, we analysed the impact of microbial CK production on the microbe’s biocontrol ability. We established a causal relationship between the production of CKs by *Pseudomonas fluorescens* (*Pfl*) strain G20-18^24^,^25^ and its ability to control the infection of *Arabidopsis* by *P. syringae* pv. *tomato* DC3000 (*Pto*) through comparisons with G20-18-derived loss-of-function and gain-of-function strains in a leaf infiltration assay. Analyses of *Arabidopsis* mutant lines impaired in defence or hormone signalling pathways revealed the necessity of functional CK perception in combination with SA defence signalling and a potential minor impact of ET, JA signalling as well as camalexin accumulation to fully establish microbial CK-mediated biocontrol. These data provide the basis for a novel microbe-based concept of biocontrol.

## Results

### Microbial CKs mediate G20-18 biocontrol

Since the CK-producing PGP *Pfl* strain G20-18 had not been tested for its biocontrol abilities, we first examined its biocontrol potential in the *Arabidopsis*–*Pto* pathosystem^26^ in comparison to its CK-deficient transposon mutants CNT1 and CNT2^24^,^25^. As CKs have been demonstrated to induce defence responses or resistance against (hemi)biotrophic foliar pathogens when applied to leaves of *Arabidopsis*^10^,^11^,^27^, rice^13^,^16^ and tobacco^12^,^28^, we decided to analyse the biocontrol potential of the *Pfl* strains when directly applied to *Arabidopsis* leaves by infiltration of cell suspensions 48 h prior to *Pto* infection. The leaf infiltration assay widely used in model pathosystems was chosen to allow us to relate the findings to the well-established immunity-relevant CK functions in leaf tissues. Although approaches such as spray inoculation or application to the root system would address more natural scenarios of interaction, they would contribute additional sources of interference with CK-mediated immunity responses, and thus, further complicate the analyses of a potential role of CK in biocontrol.

**Figure 1:**
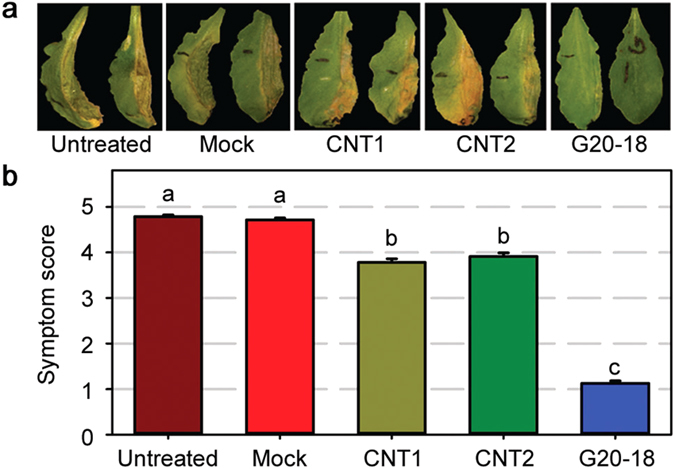
*Pfl* G20-18 suppresses *Pto* symptoms in *Arabidopsis*. (**a**) *Pto* symptom development in *Arabidopsis* leaves (right halves) 4 days post infection (dpi) with 10^6^ cfu ml^−1^ is strongly suppressed by G20-18 compared to controls and CNT pre-treatments. (**b**) Average *Pto* symptom score in *Arabidopsis* 4 dpi with 10^6^ cfu ml^−1^ is significantly lower after G20-18 pre-treatment compared to controls and CNT pre-treatments. Data are means ± s.e. n ≥ 300, letters indicate different significance groups (*P* < 0.05).

Pre-treatment with *Pfl* G20-18 heavily suppressed *Pto* symptom development at 4 days post infection (dpi), resulting in maintenance of tissue integrity, an important beneficial aspect of biocontrol applications in sustaining biomass yield. Mock pre-treatment had no effect on *Pto* symptoms compared to control infections without pre-treatment (Fig. 1a). Thus, G20-18 is considered an efficient strain for biocontrol of *Pto* in *Arabidopsis* in the leaf infiltration assays. In comparison to G20-18, both CNT transposon mutants had only a slight suppressive effect on *Pto* symptom development (Fig. 1a). The quantification of the average symptom scores over all experiments further demonstrates this biocontrol effect: G20-18 pre-treatment efficiently suppressed *Pto* symptoms by approximately 75%, CNT pre-treatments suppressed *Pto* symptoms only by 15 to 20% compared to untreated and mock controls, indicating that the CK-deficient mutants were significantly less effective than G20-18 (Fig. 1b). This highly reduced effect of the CK-deficient CNT transposon mutants on *Pto* symptom development strongly supports a role for microbial CK production in the biocontrol ability of G20-18.

As the CNT transposon mutants were generated by undirected mutagenesis via the introduction of the *TnphoA* transposon into G20-18 and were selected based on CK deficiency without detailed genetic characterization^24^, we analysed the only known CK biosynthetic gene in *Pfl* strains, *tRNA delta(2)-isopentenylpyrophosphate transferase* (*miaA*). Using primers based on known *Pfl miaA* sequences the gene was amplified from G20-18 and sequenced (Supplementary Fig. 1). Size comparison of full-length *miaA* amplicons of G20-18 and the CNT transposon mutants as well as sequence analysis ruled out *miaA* as the direct target of *TnphoA*. Subsequent semi-quantitative RT-PCR analysis revealed that *miaA* transcript levels in the CNT transposon mutants were strongly reduced by approximately 50% compared to G20-18 (Supplementary Fig. 2). This suggests that regulatory components in the CNT mutants were affected by the transposon mutagenesis, potentially interfering with *miaA* transcription or the processing and stability of *miaA* transcripts. Since the mechanism of transcriptional regulation of *miaA* is not elucidated, we used directed functional approaches to further substantiate the link between *miaA* as a determinant of microbial CK production and subsequent biocontrol activity against *Pto*.

**Figure 2:**
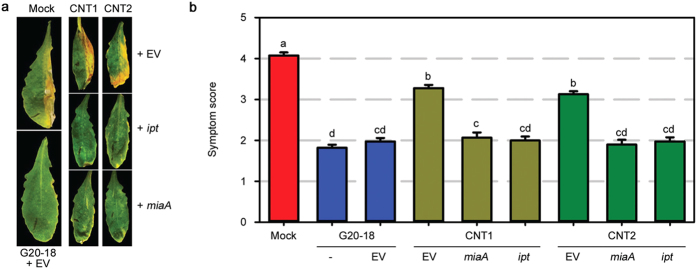
Complementation of the CNT transposon mutants with a functional CK biosynthetic gene restores their biocontrol ability. (**a**) The biocontrol ability of CNT transposon mutants is restored by complementation with functional *Atipt* or G20-18*miaA* evident from strongly reduced *Pto* symptoms (right leaf halves) 4 days post infection (dpi) with 10^6^ cfu ml^−1^. Transformation with the empty vector *pBBRMCS-5* (EV) has no effect. (**b**) Average *Pto* symptom score in *Arabidopsis* 4 dpi with 10^6^ cfu ml^−1^ after indicated pre-treatments. Data are means ± s.e. n ≥ 226, letters indicate different significance groups (*P* < 0.05).

Considering the reduced *miaA* transcript levels in the CNT transposon mutants, compared to G20-18, as the cause for the difference in biocontrol efficacy, functional complementation for CK production by the CNT transposon mutants (gain-of-function) was performed to assess the possible restoration of their biocontrol ability. Therefore, the CK biosynthetic genes *isopentenyltransferase* from *Agrobacterium tumefaciens* (*ipt*) for heterologous expression and the endogenous *Pfl* G20-18 *miaA* for homologous expression were fused to a lac-promoter in the expression vector *pBBR1MCS-5*. The different *Pfl* strains were transformed with these gain-of-function constructs or the empty vector (EV) and analysed for their biocontrol activities. The presence of the EV did not affect biocontrol activity of G20-18 as this strain efficiently restricted *Pto* symptom development (Fig. 2a) comparable to G20-18 wild-type (Fig. 2b). Also in the CNT transposon mutants, the EV did not cause changes (Fig. 2a) as symptoms were still significantly less suppressed compared to G20-18 (Fig. 2b). In contrast, the *ipt*- or *miaA*-complemented CNT transposon mutants, exhibited restored biocontrol activities as evidenced by a strong suppression of *Pto* symptom development (Fig. 2a), comparable to G20-18 biocontrol activity (Fig. 2b). This wild-type-like biocontrol activity in the two CNT transposon mutants functionally complemented via restored CK production by two different CK biosynthetic genes supports the role of microbial CKs as a key determinant for efficient biocontrol of *Pto*.

**Figure 3:**
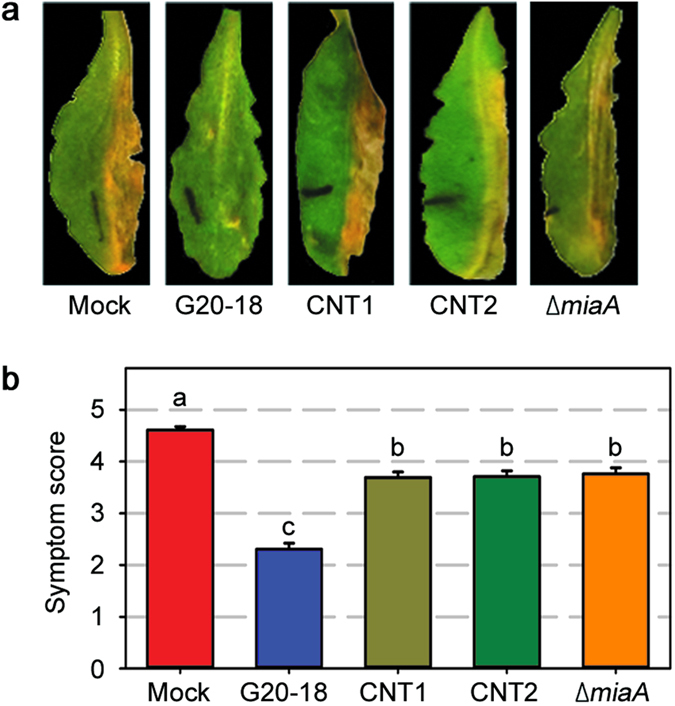
Distinct ∆*miaA* knockout in *Pfl* G20-18 exhibits a reduced biocontrol activity. (**a**) Δ*miaA* loss-of-function mutant is impaired in its biocontrol ability indicated by stronger *Pto* symptom development (right leaf halves) 4 days post infection (dpi) with 10^6^ cfu ml^−1^ compared to *Pfl* G20-18 pre-treatment. (**b**) Average *Pto* symptom score in *Arabidopsis* 4 dpi with 10^6^ cfu ml^−1^ after indicated pre-treatments. Data are means ± s.e. n ≥ 79, letters indicate different significance groups (*P* < 0.05).

To substantiate the gain-of-function data, a complementary loss-of-function approach was followed, addressing the function of *miaA* and subsequent CK production in G20-18-mediated biocontrol of *Pto*. To this end, the impact of directed knockout of the G20-18 *miaA* gene by insertion of a kanamycin resistance cassette into the *miaA* coding region on the biocontrol ability was assessed. This resulted in the *Pfl* knockout mutant Δ*miaA*, which tested PCR-positive for the integration of the disrupted *miaA* gene sequence in its genome. RT-PCR confirmed the lack of *miaA* transcripts and thus the functional knockout in this strain (Supplementary Fig. 2). Assays with this Δ*miaA* knockout mutant revealed a significant reduction in biocontrol compared to G20-18 wild-type as illustrated by stronger *Pto* symptom development (Fig. 3). Together, the gain-of-function and directed loss-of-function approaches prove the importance of microbial CK production for their biocontrol ability in the leaf infiltration assays. Interestingly, the distinct functional *miaA* knockout in Δ*miaA* (Supplementary Fig. 2) did not further reduce the biocontrol ability compared to the transposon mutants CNT1 and 2 (Fig. 3) in which low levels of *miaA* transcripts were still detectable (Supplementary Fig. 2). This suggests that the described biocontrol effect depends on minimum threshold levels of *miaA* transcripts which subsequently determine CK levels that suffice to induce resistance under particular conditions.

### G20-18 biocontrol affects CKs *in planta*

Based on the established link between *Pfl* G20-18 CK production and its biocontrol abilities described above, the *in planta* CK levels were analysed as these should ultimately reflect their contribution to the induction of resistance or defence responses^10^,^11^,^12^,^13^,^14^,^15^,^27^. Therefore, we analysed the accumulation of 25 individual CK species comprising the free nucleobases as well as conjugates^29^ in pooled samples of whole *Arabidopsis* leaves 48 h post infiltration with the different *Pfl* strains, which corresponds to the time-point of *Pto* infection. Thus, these samples integrate all processes related to each individual pre-treatment and determine the plant tissue status at the critical time-point of infection that defines the outcome of the plant-pathogen interaction. CKs were analysed in two sample sets, one comparing the pre-treatments with G20-18, the *miaA*- or *ipt*-complemented CNT transposon mutants, and mock control (Table 1 and Supplementary Table 1), and the second comparing pre-treatments with G20-18, the CNT transposon mutants, the Δ*miaA* knockout mutant, and mock control (Table 1 and Supplementary Table 2). Eight of ten CK levels that increased after G20-18 treatment in the first set (Supplementary Table 1) also increased in the second set (Supplementary Table 2). A clear trend of lower CK levels in plant tissue pre-treated with loss-of-function CNT transposon or Δ*miaA* knockout mutants was observed compared to G20-18 (ratios of 0.82 to 0.89). In contrast, this effect was reversed in tissue treated with the functionally complemented CNT transposon mutants that showed even higher CK levels compared to G20-18 (ratios of 1.08 to 1.11, Table 1). Since CK types differ in their biological activity and signalling function, the individual consideration of specific CK species is important. Total tZ-, cZ-, DHZ- and iP-type CK levels showed similar trends as total CK levels with lower levels after treatments with the CK-deficient mutants (Supplementary Table 2) and reversion in the functionally complemented CNT transposon mutants (Supplementary Table 1), which correlates with their differential effect on *Pto* symptom development (Fig. 1, 2, 3). Similarly, levels of the free nucleobases as the most active CK species^5^ were lower in tissue treated with CK-deficient mutants (ratios of 0.63 to 0.97) and higher in tissue treated with the functionally complemented CNT transposon mutants (ratios of 1.34 to 2.38) compared to G20-18 (Table 1). In particular, the individual nucleobases tZ, cZ, and iP accumulated to higher levels after treatment with the functionally complemented CNT transposon mutants compared to G20-18 (ratios of 1.30 to 2.50), while these nucleobases in general showed lower accumulation after mutant treatments (Table 1). The most prominent differences were detected in the accumulation of the highly active tZ. Treatment with the three CK-deficient mutants caused significantly lower tZ levels (ratios of 0.47 to 0.60) than G20-18 treatment (Table 1), which can directly be related to the defects in *miaA* expression as *miaA* has been identified to be responsible for the specific formation of tZ and derivatives from tRNA in different bacteria^30^,^31^,^32^. Intriguingly, exogenously supplied tZ efficiently restricted infections of *Arabidopsis* with *Pto*^10^ and tobacco with *P. syringae* pv. *tabaci* (*Pst*)^12^,^28^, while cZ had a much weaker effect on the resistance against *Pst*^28^ and iP treatment did not increase the resistance of rice against *Magnaporthe oryzae*^13^. This could explain why increased cZ or iP levels in some samples after treatment with the CK-deficient mutants had no effect on resistance against *Pto*, and emphasizes the role of tZ levels as a key parameter in G20-18-mediated biocontrol.

The analyses of a large set of individual CK levels revealed subtle though distinct changes in the host plant. In both datasets specific differences between G20-18 and its derivatives were successfully monitored and appeared to be robust even against variable background levels indicated by the variable CK levels in the control samples (Supplementary Table 1 and Supplementary Table 2). The complex regulation of CK levels in *Arabidopsis* depends on nine biosynthesis and seven catabolism genes that are potentially affected by *Pfl*, similar to other *Arabidopsis*-microbe interactions (eFP browser^33^), which could be responsible for the different CK ratios between G20-18-treated and control samples in the two sets in addition to microbial CK production (Supplementary Table 1 and Supplementary Table 2). In addition, the infiltration process, ambient conditions, inter-conversions and transport of CKs contribute to complex spatiotemporal dynamics at the cellular level, which are difficult to resolve by CK determination in plant tissue. Considering the known activity of CKs at low concentrations, the subtle differences caused by bacterial CK production linked to the CK-mediated plant defence ensures minimal interference with general plant CK homeostasis and thus minimal perturbation of other plant processes.

### G20-18 biocontrol limits pathogen growth

**Figure 4:**
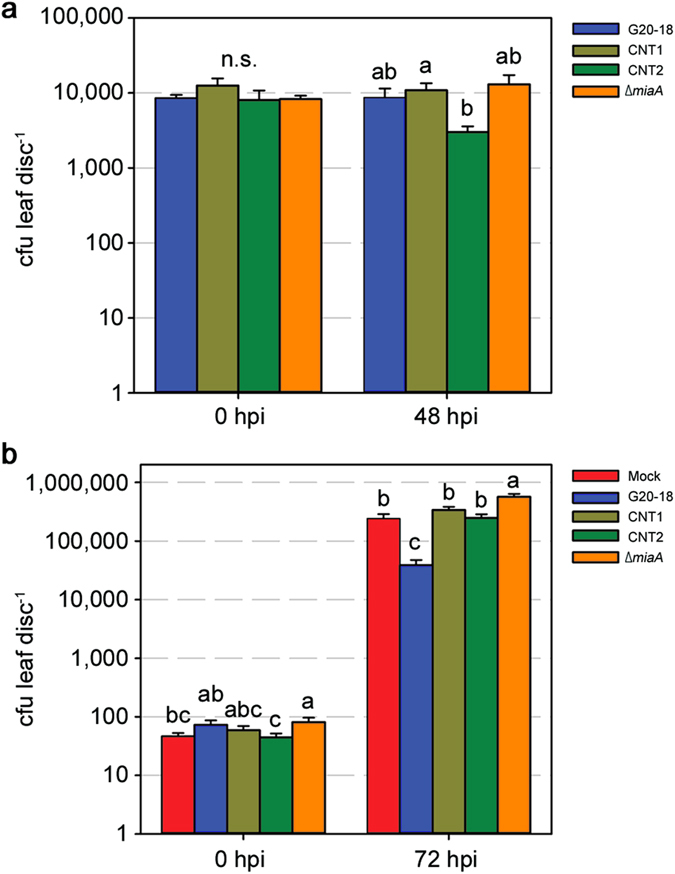
*Pfl* G20-18 and its CK-deficient mutants do not differ in growth, but differentially affect *Pto* proliferation *in planta*. (**a**) Number of viable *Pfl* cells in *Arabidopsis* leaves 0 hours post infiltration (hpi) with 10^7^ cfu ml^−1^ and at the time-point of *Pto* infection (48 hpi). n = 27. (**b**) Number of viable *Pto* cells harbouring *pMP4662* in *Arabidopsis* leaves directly after infiltration (0 hpi) with 10^5^ cfu ml^−1^ and at 72 hpi. n = 18. Data are means ± s.e., letters indicate different significance groups (*P* < 0.05).

The differential efficacy in biocontrol by G20-18 and its CK-deficient mutants could result from growth variations of the *Pfl* strains *in planta*, since the number of living *Pfl* cells may determine biocontrol by competition with *Pto* for nutrients and space^34^,^35^. Such growth differences could depend on their capacity to produce CKs, which may interfere with bacterial quorum sensing^36^ or the communication between microbe and plant for successful niche establishment^37^. Additionally, growth defects could be caused by pleiotropic effects of the applied mutagenesis unrelated to the CK deficiency, while reduced viability of the *Pfl* mutants could cause lower CK production. Therefore, the number of viable *Pfl* cells *in planta* at the time-point of *Pto* infection - 48 hours post infiltration (hpi) of *Pfl* -, was determined for G20-18 and the different mutant strains (Fig. 4a). Similar numbers of viable cells were determined directly after the infiltration (0 hpi) and at 48 hpi for all strains except CNT2 for which viable cells decreased (significantly compared to CNT1). Based on these data, growth differences between G20-18 and the analysed mutant strains can be excluded as the cause of the variations in their biocontrol abilities.

As CKs can directly contribute to a favourable physiological status by modulating primary metabolism^8^,^9^ and thus potentially affect tissue integrity, suppression of symptom development during CK-mediated resistance does not necessarily correlate with restriction of pathogen growth^28^, which is a direct result of increased resistance. To discriminate between increased resistance induced by G20-18-derived CKs and general impact on tissue integrity, we determined *Pto* growth *in planta* after pre-treatment with G20-18 and its CK-deficient mutants. *Pto* proliferation was significantly reduced after G20-18 pre-treatment compared to the mutant and mock pre-treatments at 72 hpi (Fig. 4b) and thus restricted *Pto* proliferation can be considered as the cause for reduced symptom development in the leaf infiltration assays. Further, *Pto* proliferation was strongly negatively correlated with the tZ levels determined at the time-point of infection (Table 1) following pre-treatments with the different *Pfl* strains (ranked data, Spearman’s correlation coefficient of −0.8). G20-18 pre-treatment resulted in the lowest *Pto* proliferation and the highest tZ levels, followed by pre-treatments with the CNT transposon mutants which similarly caused lower tZ levels and higher *Pto* proliferation comparable to mock treatment, while Δ*miaA* pre-treatment resulted in the lowest tZ levels and the highest *Pto* proliferation. This correlation supports the role of specific active CKs in determining biocontrol activities by inducing defence responses that act directly on the pathogen in a dose-dependent manner, similar to resistance effects induced by exogenously applied CKs^11^,^12^, which in a certain range can act in a dose-dependent manner and require specific threshold levels to be active.

### G20-18 biocontrol requires plant pathways

**Figure 5:**
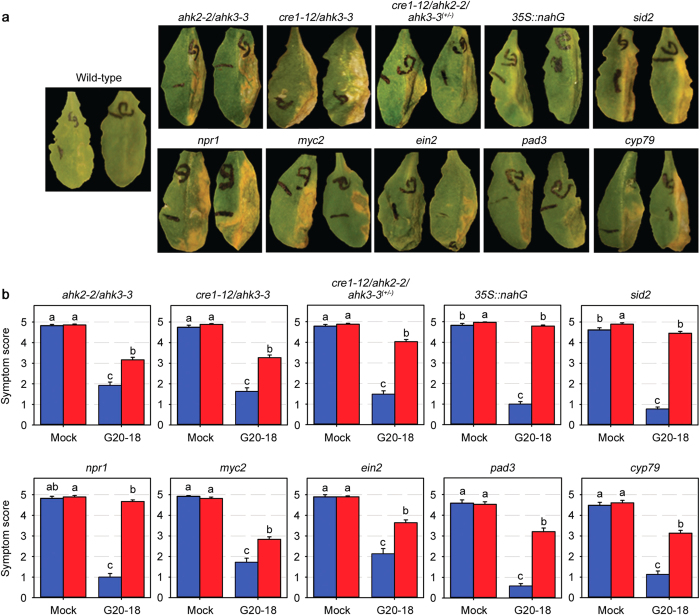
*Pfl* G20-18 biocontrol depends on functional hormonal and defence pathways of the host. (**a**) *Pto* symptom development (right leaf halves) 4 days post infection (dpi) with 10^6^ cfu ml^−1^ in indicated *Arabidopsis* lines after *Pfl* G20-18 pre-treatment. (**b**) Average *Pto* symptom scores 4 dpi with 10^6^ cfu ml^−1^ in indicated *Arabidopsis* mutant or transgenic lines (red bars) compared to Col-0 (Col-gl for *myc2*) wild-type (blue bars) pre-treated with *Pfl* G20-18 or the appropriate mock. Data are means ± s. e. n ≥ 28, letters indicate different significance groups (*P* < 0.05).

*Pfl* G20-18 showed suppressive effects on *Pto* symptom development and multiplication in *Arabidopsis* indicating direct activation of plant defences, which were lacking after pre-treatment with CK-deficient *Pfl* mutants. To dissect the underlying plant mechanisms, the efficiency of G20-18-mediated biocontrol was determined in several *Arabidopsis* lines impaired in phytohormone and/or defence-related mechanisms (Fig. 5a). Since we identified microbial CK production as a determinant of *Pfl* G20-18-mediated biocontrol against *Pto*, we assumed functional CK perception as the initial step of CK signalling in the plant to be essential. In *Arabidopsis* CK perception depends on the three membrane-bound histidine kinases AHK2, AHK3 and AHK4/CRE1/WOL^6^. The function of these *Arabidopsis* CK receptors in G20-18 biocontrol was assessed in the double mutant lines *ahk2-2*/*ahk3-3* and *cre1-12*/*ahk3-3*, and the triple mutant *cre1-12*/*ahk2-2*/*ahk3-3*^(+/−)^ (homozygous for *cre1-12* and *ahk2-2*, heterozygous for *ahk3-3*)^38^. G20-18-mediated biocontrol was reduced in all three mutant lines (Fig. 5a), illustrated by significantly elevated *Pto* symptom development compared to the wild-type Col-0 (Fig. 5b). This indicates that all three receptors function as signalling components of CK-dependent biocontrol by G20-18, which is supported by the finding that the triple mutant exhibited the strongest defect (Fig. 5b). However, a rudimentary G20-18 biocontrol effect is still observed in these mutant plant lines, which is either due to residual CK perception or is unrelated to G20-18 CK production and/or plant CK signalling.

SA was demonstrated as a key central defence signalling component of CK-mediated resistance, mainly depending on NPR1 signalling, against *Pto* in *Arabidopsis*^10^, but also as a parameter of CK-induced resistance or defence responses in other plant species^12^,^13^,^28^. The role of SA in G20-18-mediated biocontrol was assessed in *Arabidopsis* lines either overexpressing *nahG* (*35S::nahG*), a SA-degrading enzyme from *Pseudomonas putida*^39^, or defective in SA biosynthesis (*sid2*)^40^ or SA signalling (*npr1*)^41^. In agreement with the known SA-dependent tZ-mediated resistance effect in *Arabidopsis*^10^, G20-18 pre-treatment was almost completely ineffective in these lines as *Pto* symptoms were not suppressed (Fig. 5a). *35S::nahG*, *sid2*, and *npr1* (Fig. 5b) showed *Pto* symptoms after G20-18 treatment comparable to the mock treatment in the plant mutants and Col-0 wild-type, hence SA accumulation as well as functional SA signalling have to be considered as major parameters in CK-mediated biocontrol.

To examine involvement of the defence-related phytohormones JA and ET, which are important for inducing ISR as part of biocontrol and for priming effects mediated by beneficial microbes^4^,^20^, G20-18 biocontrol assays were performed in the mutant lines *myc2* (*jin1*)^42^ which is partially insensitive to JA^43^, and *ein2*^44^, which is insensitive to ET. In both *Arabidopsis* lines, the suppressive effect of G20-18 on *Pto* symptoms was reduced (Fig. 5a). Although this reduction was significant compared to *Arabidopsis* wild-type Col-gl (*myc2*) and Col-0 (*ein2*; Fig. 5b), it is considerably lower than observed in SA-related plant mutants, indicating a minor role of JA and ET in this biocontrol mechanism in leaf infiltration assays. As phytoalexins also potentially contribute to biocontrol effects as antimicrobial compounds^45^,^46^, G20-18 biocontrol effects were analysed in *Arabidopsis pad3*^47^ and *cyp79*^48^ mutants deficient in camalexin, the key phytoalexin in *Arabidopsis*^49^. In both lines, the effect of G20-18 on *Pto* was reduced, as evidenced by stronger symptom development compared to Col-0 (Fig. 5a), which was significantly lower, however, than the mock controls (Fig. 5b). These data suggest a minor role of camalexin in G20-18-mediated biocontrol in the leaf infiltration assays, which possibly depends on microbial CKs similar to CK-induced resistance effects shown in tobacco^12^,^14^,^15^.

## Discussion

The biological control of plant diseases by beneficial microbes includes well-known mechanisms such as antibiotic production, competition for nutrients and space, or activation of plant defences^34^,^35^. Our results identified *Pfl* G20-18 as a biocontrol strain that efficiently suppressed *Pto* infection of *Arabidopsis* when applied to leaves. By dual functional approaches modulating either CK production in the beneficial microbe or CK perception and defence signalling in the host plant, we, to the best of our knowledge, for the first time, identified CK as a microbe-derived phytohormone that functions as a key determinant of microbial biocontrol activity by directly activating plant resistance. This novel concept seems to be independent of classic biocontrol mechanisms such as competition or antibiotic production, and strongly depends on functional CK perception as well as SA accumulation and signalling of the host plant (Fig. 5). This is in agreement with known CK-SA interactions in plant immunity^10^,^12^,^13^, particularly considering the strong dependency on SA of CK-induced resistance against *Pto* in *Arabidopsis* leaves by exogenously applied tZ^10^. In contrast, the CK-mediated biocontrol of G20-18 in *Arabidopsis* seems to depend not or only to a limited extent on JA, ET and camalexin accumulation. The apparent minor effect of these defence signalling pathways could be due to the specific biocontrol assays performed in *Arabidopsis* leaves and particularly the contribution of JA and ET is probably more relevant in natural microbe-root interactions. Furthermore, these mechanisms do not necessarily depend on microbial CK production and could therefore be responsible for the rudimentary suppression of *Pto* symptoms caused by the CK-deficient *Pfl* mutants. Generally, the contribution of all these underlying, networked mechanisms to CK-mediated biocontrol as well as the relevance of microbial CKs for biocontrol *per se*, can vary depending on the conditions of the interaction between the beneficial microbe and the plant. Based on the presented results, which strongly support the potential of microbial CKs for biocontrol effects, directed analyses of CK function in more complex biocontrol systems such as the natural interaction between beneficial microbes and plant roots or their relevance in practical applications such as spraying or seed coating can be conducted in the future.

In the model biocontrol assays used here G20-18 seems to cause only subtle and fine-tuned, but highly efficient changes in the host plant CK levels. Induction of the subsequent resistance seems to be specifically regulated, probably in concert with additional mechanisms such as direct modulation of the metabolic plant-pathogen interface^50^ or interference with the niche establishment of *Pto*^37^. Since CKs are critically involved in various aspects of plant growth, development and physiology^6^, such a highly fine-tuned mechanism ensures minimal perturbation of other central plant processes and thus excludes any kind of dramatic impact apparent in transgenic plants with modulated CK homeostasis^51^,^52^,^53^, or known from plant diseases related to microbial CK production, such as tumours caused by *Agrobacterium*^54^,^55^. This aspect of fine-tuned CK modulation is of particular importance in the context of plant immunity, as exogenously supplied CKs (benzyl adenine or tZ-riboside) below certain threshold concentrations were shown to potentially also increase susceptibility in the interaction of *Arabidopsis* with *H. arabidopsidis*^11^ and *Pto*^56^ under the specific experimental conditions used in these studies. In the *Arabidopsis*-*Pto* interaction the activity of the *Pto* effector HopQ1 activated CK signalling, had an impact on some CK levels, and interfered with immunity^56^. Our results together with the increasing experimental evidence within other reports on resistance mediated by increased CK levels emphasize that CK modulation of plant immunity has to be fine-tuned to cause beneficial effects for the plant and that the outcome is influenced by additional parameters.

Considering the assumed widespread ability of microbes to produce CKs based on the presence of CK biosynthetic genes, it may be concluded that this novel CK-dependent defence mechanism also contributes to other yet uncharacterized biocontrol systems, potentially including positive effects on environmental stress tolerance as indicated for CKs^57^ and CK-producing microbes^23^. Thus, the possibility to achieve abiotic and biotic co-tolerance via microbial CK production is a potential practical application of the results obtained, provided that they are validated in the biocontrol and application systems of interest. The positive effects mediated by beneficial microbes, such as PGP, increased tolerance to environmental stress and pathogen resistance might be integrated by their capacity to produce CK profiles of specific quantity and composition. Thus, screening for microbial phytohormone, particularly CK production must be considered as an additional parameter when characterizing new beneficial plant-microbe interactions or potential biocontrol strains. This finding that microbial CKs determine biocontrol effects could potentially offer novel options for developing alternative strategies for integrated plant protection combined with PGP. Both effects contribute to improved plant productivity, which is needed to cope with challenges such as increasing world population, climate change and restricted use of classic pesticides. Optimized CK production by beneficial microbes could be an excellent biological alternative to classic pesticides and fertilizers, and may work efficiently in different (crop) plant species since CK-dependent resistance mechanisms have been identified in a variety of plants.

## Methods

### Plant lines and growth conditions

*Arabidopsis* plants were grown in soil under controlled short day (8/16 h, 22/20 °C day/night) conditions at 60% relative humidity in growth chambers (APT.line™ KBW 720, BINDER GmbH), and were used for experiments approximately 6 weeks after germination as described before^58^. Genetically modified plant lines were cultivated in proximity to the appropriate wild-type in the same trays (10 pots per tray) to avoid position effects.

*Arabidopsis* wild-type Col-0 was used for all standard experiments and as comparison for genetically modified lines, except *myc2* (Col-gl background). Plant components functionally involved in the described biocontrol mechanism were identified by testing appropriate established genetically modified *Arabidopsis* lines in biocontrol assays. The relevance of CK perception was analysed in the *Arabidopsis* CK-receptor mutant lines *ahk2-2*/*ahk3-3*, *cre1-12*/*ahk3-3*, and *cre1-12*/*ahk2-2*/*ahk3-3*^(+/−)^ (homozygous for *cre1-12* and *ahk2-2*, heterozygous for *ahk3-3*). The role of SA was analysed in the SA-deficient lines *35S::nahG* (overexpressing SA-degrading SA-hydroxylase from *P. putida*), *sid2*, and the SA signalling mutant *npr1*. Involvement of ET and JA was analysed in the ET insensitive line *ein2*, and the JA insensitive line *myc2*. The camalexin-deficient lines *cyp79* and *pad3* were analysed to determine the role of this *Arabidopsis* key phytoalexin.

### Bacterial strains

The virulent hemibiotrophic bacterial pathogen *Pseudomonas syringae* pv. *tomato* DC3000 (*Pto*) was used for all infections as described before^58^. For determination of *Pto* proliferation *in planta* following pre-treatments with *Pseudomonas fluorescens* (*Pfl*) strains, *Pto* was transformed with *pMP4662*^59^ to facilitate additional selection against tetracycline which is necessary to avoid unspecific background (co-cultivation) by spontaneous rifampicin resistance of *Pfl* cells^24^. Freshly grown (28 °C, 200 rpm) *Pto* cells from liquid cultures in 50 ml LB medium containing 50 mg l^−1^ rifampicin (and 20 mg l^−1^ tetracycline for the *pMP4662* transformed strain) were pelleted, re-suspended in 10 mM MgCl_2_ and adjusted to the desired concentration for the experiments using the BioPhotometer plus (Eppendorf AG).

*Pfl* G20-18 was tested for its biocontrol ability and subsequently used for biocontrol assays. It was tested against its transposon (*TnphoA*) mediated CK-deficient mutants CNT1 and CNT2^24^, CNT transposon mutants functionally complemented with functional CK biosynthetic genes (homologous expression of G20-18*miaA* or heterologous expression of *Agrobacterium tumefaciens ipt* [*Atipt*]) in *pBBRMCS-5* and a loss-of-function mutant of G20-18 with a distinct disruption of its CK biosynthetic gene *miaA* (∆*miaA*). A detailed description of the cloning procedure and generation of *Pfl* derivatives are available as Supplementary Methods. The different *Pfl* strains were cultivated in 50 ml LB medium (28 °C, 200 rpm) containing 10 μM adenine^24^ and appropriate antibiotics: 50 mg l^−1^ ampicillin for G20-18; 50 mg l^−1^ ampicillin and 20 mg l^−1^ gentamycin for G20-18 transformed with *pBBRMCS-5*; 50 mg l^−1^ ampicillin and kanamycin for CNT1, CNT2, and ∆*miaA*; 50 mg l^−1^ ampicillin, kanamycin, and 20 mg l^−1^ gentamycin for CNT mutants transformed with *pBBRMCS-5* derivatives. *Pfl* cells were processed as described for *Pto* including a washing step in 30 ml 10 mM MgCl_2_ before the final resuspension.

### Biocontrol experiments

For biocontrol assays, whole *Arabidopsis* leaves were infiltrated with *Pfl* cell suspensions (OD_600_ = 0.02) or 10 mM MgCl_2_ as a mock control using a needleless syringe two days prior to infection with *Pto*. *Pto* infection was performed as described before^58^ by infiltration of *Arabidopsis* leaf halves with 10^5^ cfu ml^−1^ for *Pto* proliferation determination or 10^6^ cfu ml^−1^ for analysis of symptom development, respectively.

*Pto* symptom development (infiltrated leaf halves) was evaluated 4 days post infection (dpi) based on an adapted scale^12^ consisting of 7 categories (Supplementary Fig. 3). Viable *Pfl* cells and *Pto* proliferation *in planta* were determined similar to published procedures^60^. Discs of infiltrated leaves were excised at indicated hours post infiltration (hpi) using a cork borer of 0.4 cm diameter, ground and re-suspended in 1 ml 10 mM MgCl_2_. 100 μl of serial 1:10 dilutions were plated in triplicate on LB medium containing appropriate antibiotics for selection and colony formation was determined after 36 h incubation at 28 °C.

### Cytokinin determination

For CK determination, a minimum of 10 *Arabidopsis* leaves per sample were harvested 48 hpi with *Pfl* strains or the mock control, immediately frozen and ground in liquid nitrogen. CKs were extracted and determined by UHPLC-MS/MS as described before^29^. The CK-types quantified in this study are *cis*-zeatin (cZ), cZ-*O*-glucoside (cZOG), cZ-riboside (cZR), cZR-*O*-glucoside (cZROG), cZ-9-glucoside (cZ9G), cZR-5′-monophosphate (cZR5′MP), dihydrozeatin (DHZ), DHZ-*O*-glucoside (DHZOG), DHZ-riboside (DHZR), DHZR-*O*-glucoside (DHZROG), DHZ-7-glucoside (DHZ7G), DHZ-9-glucoside (DHZ9G), DHZR-5′-monophosphate (DHZR5′MP), isopentenyladenine (iP), iP-riboside (iPR), iP-7-glucoside (iP7G), iP-9-glucoside (iP9G), iPR-5′-monophosphate (iPR5′MP), *trans*-zeatin (tZ), tZ-*O*-glucoside (tZOG), tZ-riboside (tZR), tZR-*O*-glucoside (tZROG), tZ-7-glucoside (tZ7G), tZ-9-glucoside (tZ9G), tZR-5′-monophosphate (tZR5′MP).

### Statistical analysis

Statistical analyses were performed for datasets deriving from a minimum of three biological experiments. Unpaired Student’s t-test was used to compare group differences. P values < 0.05 were considered significant and letters in bar graphs indicate different significance groups. *, **, and *** indicate significant differences at the 0.05, 0.01, and 0.001 levels of confidence, respectively.

## Additional Information

**Accession codes:** The obtained sequence of the *Pseudomonas fluorescens* G20-18 *miaA* gene has been deposited in the GenBank database under the accession code KM593658.

**How to cite this article**: Großkinsky, D. K. *et al.* Cytokinin production by *Pseudomonas fluorescens* G20-18 determines biocontrol activity against *Pseudomonas syringae* in *Arabidopsis*. *Sci. Rep.* **6**, 23310; doi: 10.1038/srep23310 (2016).

## Supplementary Material

Supplementary Information

## Figures and Tables

**Table 1:**
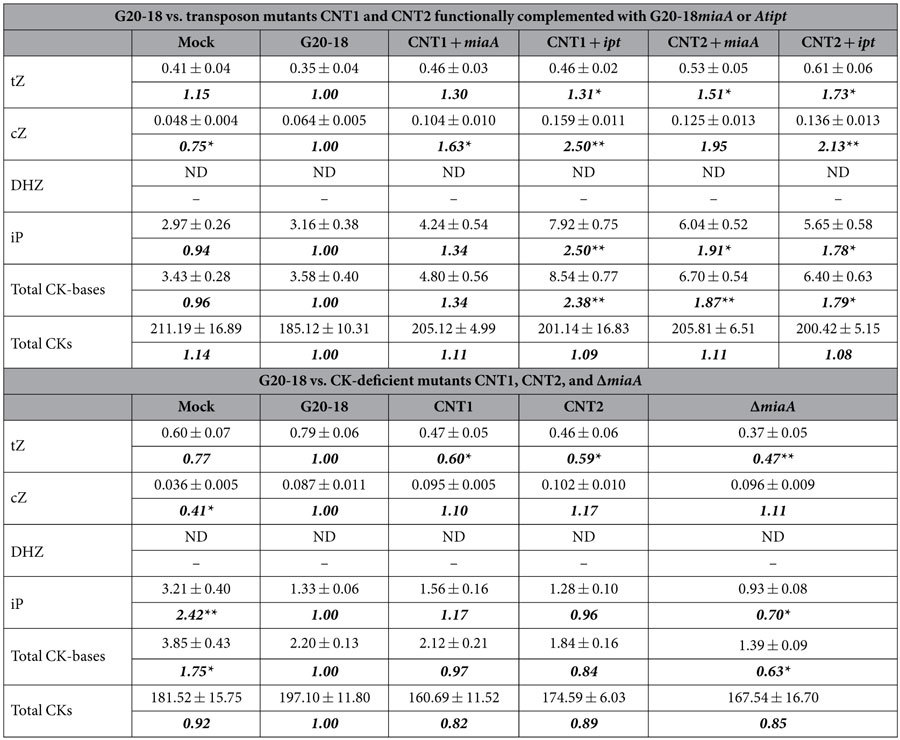
Cytokinin levels in *Arabidopsis* Col-0 48 h post infiltration with *Pfl* strains.

CK-levels in pmol g^−1^ fresh weight. Data are represented as mean ± s.e. n = 3. Ratios to G20-18 treatment are given in bold. * and ** indicate significant differences at the 0.05 and 0.01 levels of confidence, respectively. cZ, *cis*-zeatin; DHZ, dihydrozeatin; iP, isopentenyladenine; ND, not detected; tZ, *trans*-zeatin.
