# Supplementary material for: Cytokinin production by Pseudomonas fluorescens G20-18 determines biocontrol activity against Pseudomonas syringae in Arabidopsis
Source: Sci Rep. 2016 Mar 17;6:23310. doi: 10.1038/srep23310 (PMC4794740; doi:10.1038/srep23310)
Supplement: Supplementary Information [file srep23310-s1.doc]

**Supplementary Information**

**Cytokinin production by *Pseudomonas* *fluorescens* G20-18 determines biocontrol activity against *Pseudomonas syringae* in *Arabidopsis***

Dominik K. Großkinsky, Richard Tafner, María V. Moreno, Sebastian A. Stenglein, Inés E. García de Salamone, Louise M. Nelson, Ondřej Novák, Miroslav Strnad, Eric van der Graaff & Thomas Roitsch

**Supplementary Tables 1 to 3**

**Supplementary Table 1 | Cytokinin levels in *Arabidopsis* Col-0 48 h post infiltration with *Pfl* strains.**
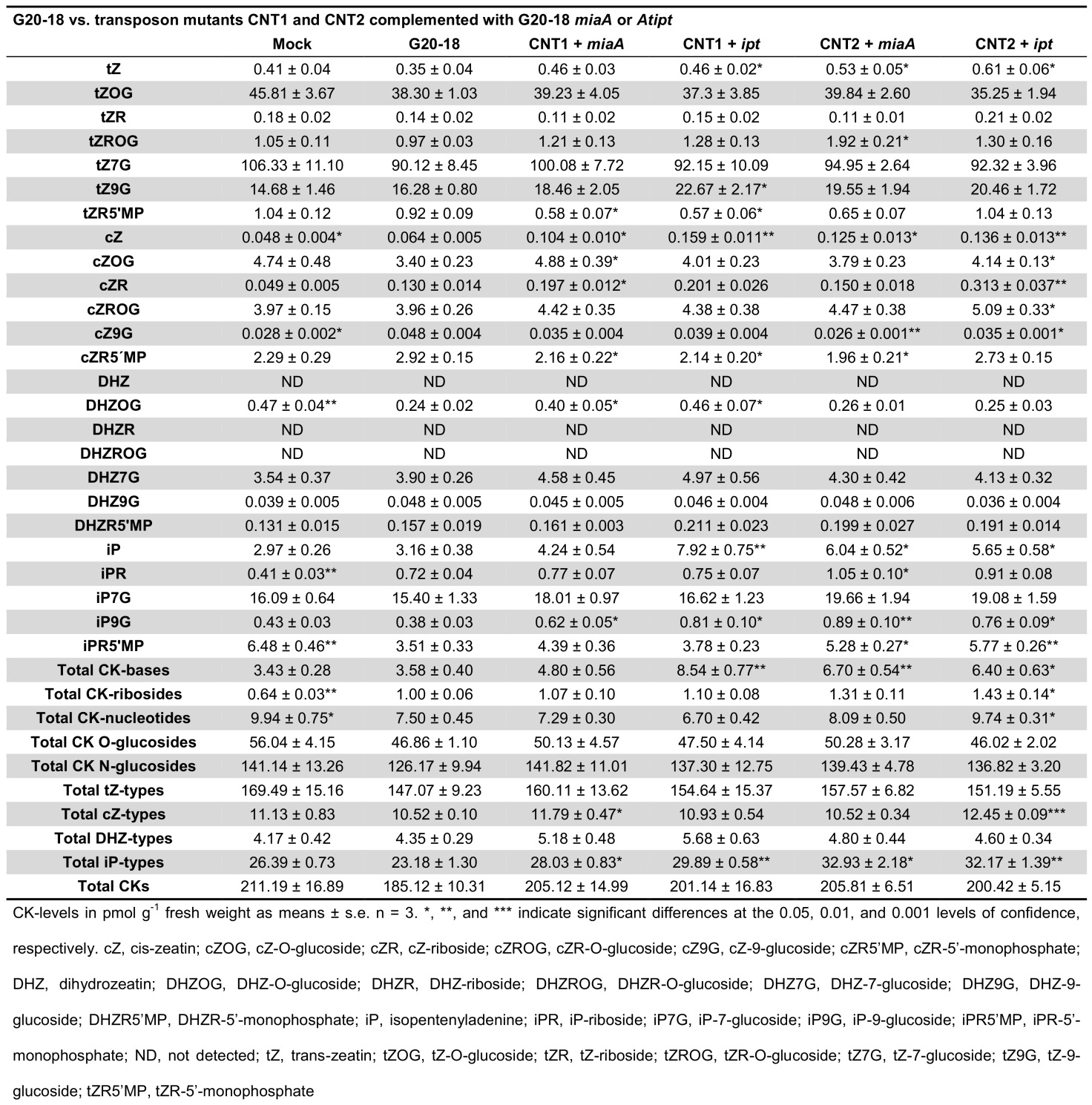


**Supplementary Table 2 | Cytokinin levels in *Arabidopsis* Col-0 48 h post infiltration with *Pfl* strains.**

**
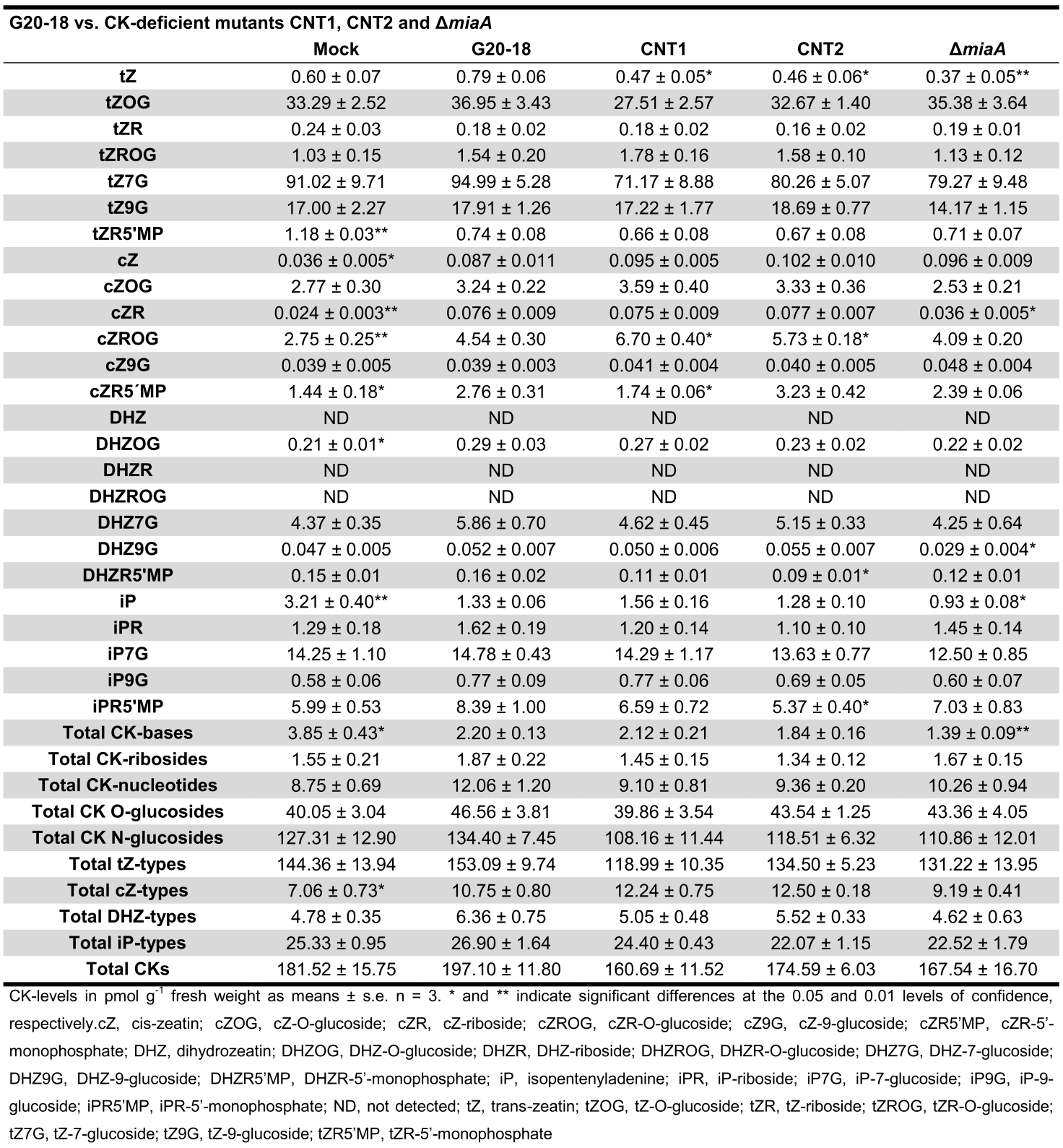
**

**Supplementary Table 3 | Primers used in this study.**

**
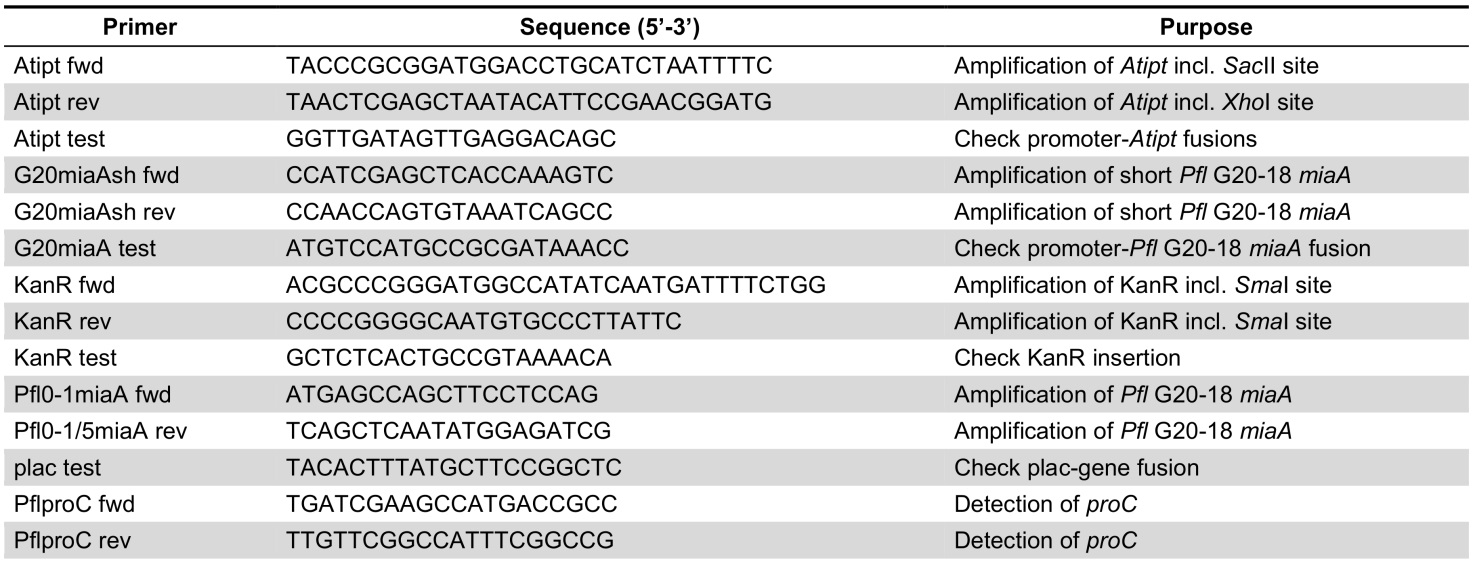
**

**Supplementary Figures 1 to 3**

**
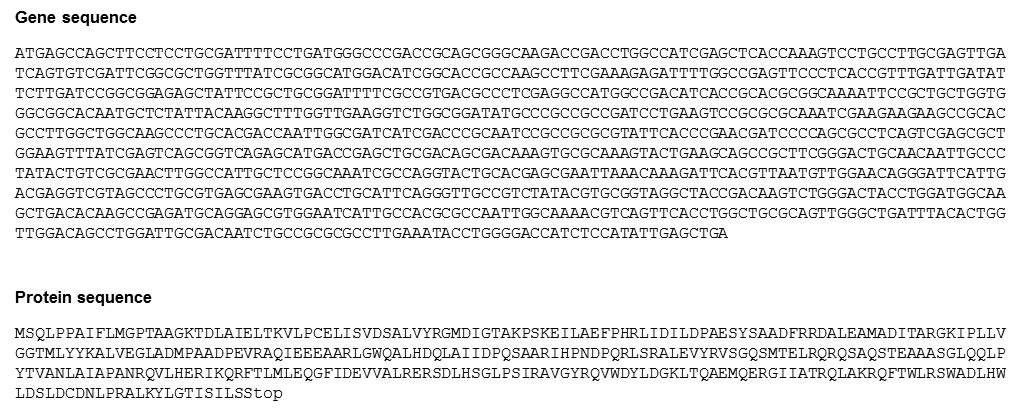
**

**Supplementary Figure 1 | Gene and protein sequence of *Pfl*G20-18 *miaA*.** Gene and protein sequence of *Pfl*G20-18 *miaA* obtained by Sanger sequencing of a cloned *miaA* amplicon in *pJet1.2* derived from genomic *Pfl*G20-18 DNA using proofreading polymerase. The DNA sequence obtained was translated to protein sequence using the ExPASy translation tool (<http://web.expasy.org/translate/>).


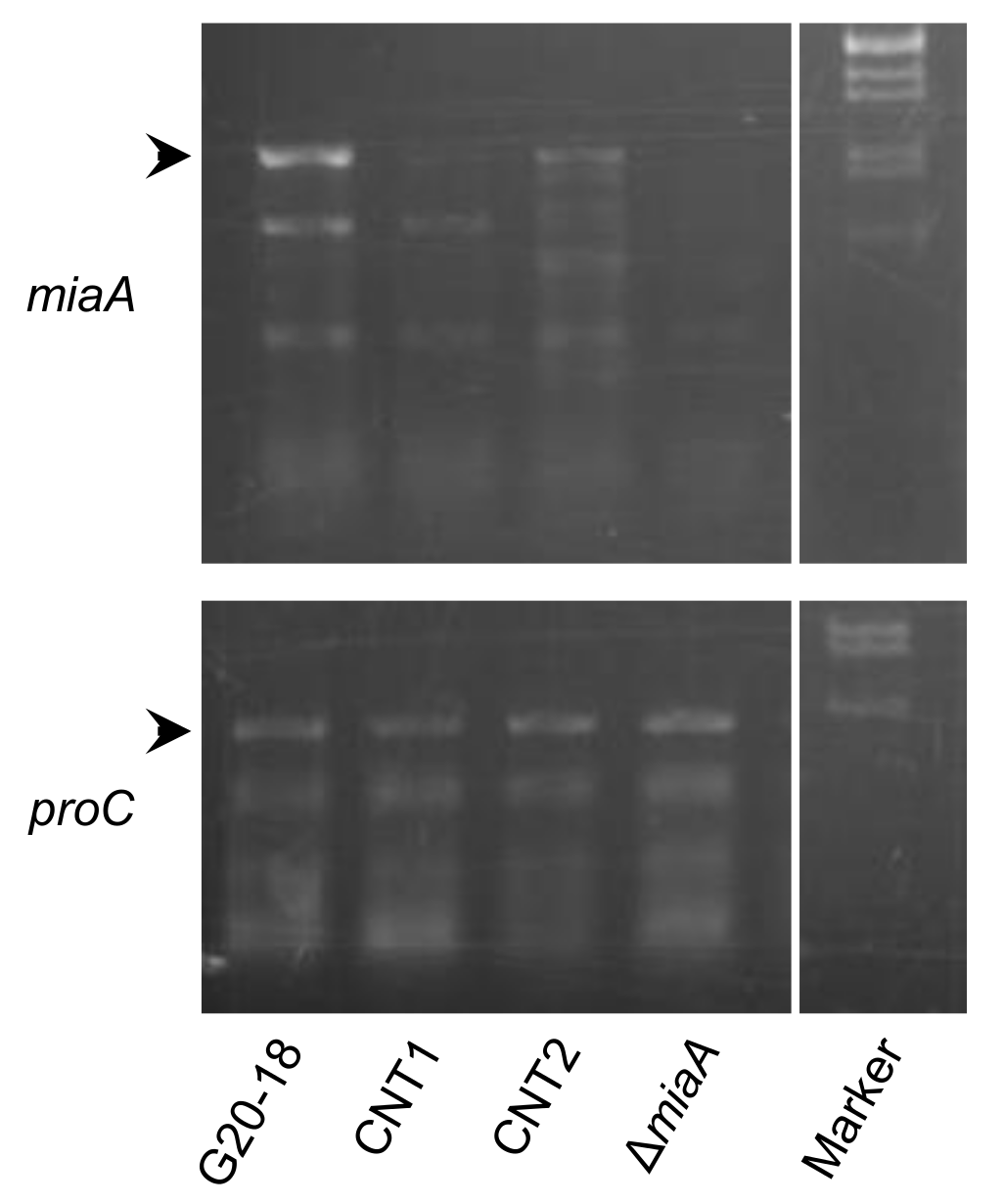
 **Supplementary Figure 2 | Transcript levels of the *Pfl*G20-18 CK-biosynthesis gene *miaA* are reduced in CNT1 and CNT2, and absent in the distinct *miaA* knockout strain.** Transcript levels of the CK biosynthesis gene *miaA* (arrow head in upper panel) in *Pfl*G20-18 normalized to *pyrroline-5-carboxylate reductase* (*proC*) transcripts (arrow head in lower panel) compared to the transposon mutants CNT1 and CNT2, and to the Δ*miaA* knockout mutant.


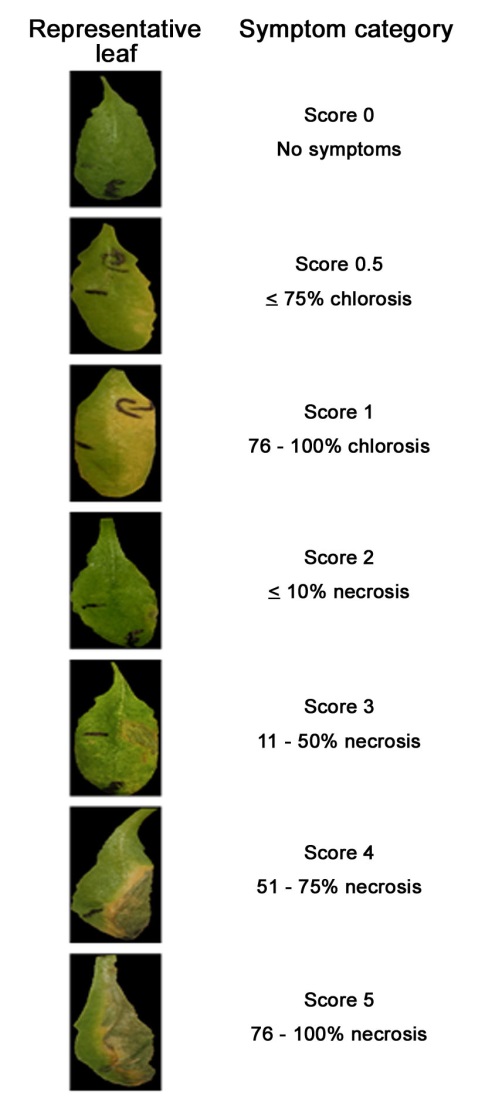


**Supplementary Figure 3 | *Pto* symptom scale.** Representative *Pto* symptoms in *Arabidopsis* leaves (right leaf halves) for the 7-category scale.

**Supplementary Methods**

**Cloning and generation of *Pfl* strains**

All cloning procedures were performed according to standard methods of the suppliers’ manuals. DNA sequences were amplified using the specific primers indicated (Sigma-Aldrich); correct insertions/fusions were identified by PCR using appropriate test primers (Supplementary Table 3) and *Taq* polymerase (Segenetic). Successfully transformed bacteria were identified by selection on LB plates containing appropriate antibiotics.

The CK biosynthetic gene *miaA* of *Pfl* G20-18 was initially amplified from genomic DNA using primers Pfl0-1miaA fwd and rev (Supplementary Table 3), which are based on the *miaA* sequence of *Pfl* strain 0-1 (accession NC_007492), and proofreading Phusion® polymerase (Thermo Scientific). The amplicon was cloned into pJet1.2/blunt (Thermo Scientific) and the 972 bp full length *miaA* sequence (Supplementary Fig. 1) was obtained by Sanger sequencing (Microsynth AG) using standard pJet1.2 sequencing primers (Thermo Scientific).

For the functional complementation of CK biosynthesis in the *Pfl* CNT transposon mutants, the *Pfl* G20-18 *miaA* gene (homologous expression) or the *Atipt*^12^ gene (heterologous expression; *Xba*I and blunted *Not*I end) was transferred to *pBBR1MCS-5*^61^ (*Xba*I and blunted *Pst*I end). CNT1 and CNT2 were transformed with the resulting vectors harboring G20-18*miaA* or *Atipt* under the control of the lac-promoter, using electroporation (2.5 kV cm^-1^ for 5.8 ms; MicroPulser™ Electroporator, Bio-Rad). Additionally, *Pfl* G20-18, CNT1 and CNT2 were transformed with the empty vector as control.

For the functional knockout of *miaA*, a shortened amplicon of 840 bp (*miaA*_65-904_) was obtained using the primers G20miaAsh fwd and rev (Supplementary Table 3) and cloned into pJet1.2/blunt. The kanamycin resistance cassette (KanR) containing *nptIII* (flanked by *Sma*I) was amplified from *pBI121*^62^ using the primers KanR fwd and rev and cloned into pJet1.2/blunt. The *Sma*I KanR-fragment was inserted at the *Afe*I site of *miaA*_65-904_ (position 433; position 497 of the full length gene). The disrupted sequence was cut with *Not*I and *Xba*I, blunted and inserted at the *Sma*I site of the mobilizable suicide vector *pK18mobGII*^63^. The resulting vector was transferred to *Pfl* G20-18 by triparental mating using *Escherichia coli* HB101 containing the helper plasmid *pRK2013*^64^ to obtain ∆*miaA*. Insertion of KanR into the genomic *miaA* was detected by PCR using the primers Pfl0-1miaA fwd or rev in combination with the primer KanR test.

The functional *miaA* knockout was confirmed by comparative detection of transcripts in *Pfl* G20-18, CNTs and ∆*miaA*. Therefore, 25 ml of LB medium with appropriate antibiotics were inoculated with 2.5 ml of *Pfl* pre-cultures and incubated at 28 °C and 200 rpm. After 90 min 10 µM adenine^24^ was added to the growing culture. 90 min later, cells from 3 ml of each culture were pelleted and total RNA was isolated using the RNAtidy reagent (AppliChem). For cDNA synthesis, RNA was reverse-transcribed using the RevertAid reverse transcriptase (Thermo Scientific) and random hexamer primers (Roth). Specific amplification (28 cycles) was performed using the primers G20miaAsh fwd and rev for the 840 bp *miaA* amplicon, and the primers proC fwd and rev for a 263 bp *pyrroline-5-carboxylate reductase* (*proC*) amplicon for normalization^65^, based on known *Pfl* *proC* sequences. Optimization of the RT-PCR and relative quantification of obtained bands was performed as previously described^12^.

**References unique to Supplementary Methods**

1. Kovach, M. E. *et al.* Four new derivatives of the broad-host range cloning vector pBBRMCS, carrying different antibiotic-resistance cassettes. *Gene* **166,** 175-176 (1995).
2. Jefferson, R. A., Kavanagh, T. A. & Bevan, M. W. GUS fusions: β-glucuronidase as a sensitive and versatile gene fusion marker in higher plants. *EMBO J.* **6,** 3901-3907 (1987).
3. Katzen, F., Becker, A., Ielmini, M. V., Oddo, C. G. & Ielpi, L. New mobilizable vectors suitable for gene replacement in gram-negative bacteria and their use in mapping of the 3' end of the *Xanthomonas campestris* pv. campestris *gum* operon. *Appl. Environ. Microbiol.* **65,** 278-282 (1999).
4. Goldberg, J. B. & Ohman, D. E. Cloning and expression in *Pseudomonas aeruginosa* of a gene involved in the production of alginate. *J. Bacteriol.* **158,** 1115-1121 (1984).
5. Savli, H. *et al.* Expression stability of six housekeeping genes: a proposal for resistance gene quantification studies of *Pseudomonas aeruginosa* by real-time quantitative RT-PCR. *J. Med. Microbiol.* **52,** 403-408 (2003).
